# Diindolylmethane suppresses ovarian cancer growth and potentiates the effect of cisplatin in tumor mouse model by targeting signal transducer and activator of transcription 3 (STAT3)

**DOI:** 10.1186/1741-7015-10-9

**Published:** 2012-01-26

**Authors:** Prabodh K Kandala, Sanjay K Srivastava

**Affiliations:** 1 Department of Biomedical Sciences and Cancer Biology Center, Texas Tech University Health Sciences Center, Amarillo, TX 79106, USA

**Keywords:** apoptosis, angiogenesis, cisplatin, diindolylmethane, STAT3

## Abstract

**Background:**

Signal transducer and activator of transcription 3 (STAT3) is activated in majority of ovarian tumors and confers resistance to cisplatin treatment in patients with ovarian cancer. We have reported previously that diindolylmethane (DIM) inhibits the growth of ovarian cancer cells. However, to date the exact mechanism by which DIM induces growth suppressive effects has not been clear. In this report the mode of action of DIM is investigated.

**Methods:**

Six human ovarian cancer cell lines and an ovarian tumor xenograft animal model were used to study the effect of diindolylmethane alone or in combination with cisplatin.

**Results:**

Diindolylmethane treatment induced apoptosis in all six ovarian cancer cell lines. Phosphorylation of STAT3 at Tyr-705 and Ser-727 was reduced by DIM in a concentration-dependent manner. In addition, diindolylmethane treatment inhibited nuclear translocation, DNA binding, and transcriptional activity of STAT3. Interleukin (IL)-6-induced phosphorylation of STAT3 at Tyr-705 was significantly blocked by DIM. Overexpression of STAT3 by gene transfection blocked DIM-induced apoptosis. In addition, DIM treatment reduced the levels of IL-6 in ovarian cancer cells and in the tumors. DIM treatment also inhibited cell invasion and angiogenesis by suppressing hypoxia-inducible factor 1α (HIF-1α) and vascular epithelial growth factor (VEGF). Importantly, diindolylmethane treatment potentiated the effects of cisplatin in SKOV-3 cells by targeting STAT3. Oral administration of 3 mg diindolylmethane per day and subsequent administration of cisplatin substantially inhibited *in vivo *tumor growth. Western blotting analysis of tumor lysates indicated increased apoptosis and reduced STAT3 activation.

**Conclusions:**

These findings provide a rationale for further clinical investigation of DIM alone or in combination for chemoprevention and/or chemotherapy of ovarian cancer.

## Background

Ovarian cancer continues to be a major worldwide gynecological malignancy. Approximately 25,000 new cases are diagnosed each year in the USA, and 15,000 patients die of this malignancy [1]. Currently, no sufficiently accurate screening tests to diagnose this malignancy are available. Consequently, it is detected only in its late stages leading to minimal survival rates after diagnosis. At stage III, ovarian cancer metastasizes and spreads to the surrounding organs such as the peritoneum and stomach. By stage IV, ovarian cancer spreads to distant metastatic organs such as the lungs and liver. Cisplatin is a well established platinum drug used to treat various cancers, including ovarian cancer [2,3]. Patients treated with cisplatin often relapse or do not respond to the treatment. In addition, at higher doses cisplatin exerts side effects such as nephrotoxicity and ototoxicity in patients [4]. Several reports suggest that signal transducer and activator of transcription 3 (STAT3) overexpression is positively associated with cisplatin resistance [5].

The STATs are a novel class of transcription factors that are positively associated with the growth and survival of cells [6]. STAT3 is a receptor tyrosine kinase that is activated either by upstream receptor kinases such as Janus activated kinases (JAKs) or cytokines such as interleukin (IL)-6 [7]. When IL-6 binds to its receptors, it activates STAT3 by phosphorylating it at Tyr-705. Activation of STAT3 at Tyr-705 leads to formation of a homodimer that translocates to the nucleus, where it binds to the promoter regions of several genes that transactivate STAT3-responsive genes such as Mcl-1, survivin and cyclin D1 [8-10]. It is also phosphorylated at Ser-727, which is not required for DNA binding activity but is important for its maximal transcriptional activity. STAT3 activates vascular endothelial growth factor (VEGF), thereby promoting neovascularization in tumors [11]. It also regulates hypoxia-inducible factor 1α (HIF-1α) and vascular epithelial growth factor (VEGF) during hypoxia, leading to hypoxia-induced angiogenesis [12,13].

Previously published reports suggest that STAT3 is overexpressed in various tumors, including ovarian tumors [10]. A recent clinical study scored 322 patients for overexpression of phosphorylated (p)-STAT3 and observed that 303 patients were positive for hyperactivation of STAT3, accounting for 94% of the study group [14]. Furthermore, various reports indicate the role of STAT3 in resistance of ovarian cancer to chemotherapy [5]. Since STAT3 is involved in various aspects of cancer growth ranging from tumor initiation, angiogenesis, and metastasis, it represents an attractive target for intervention.

3,3'-Diindolylmethane (DIM), an active metabolite of indole-3-carbinol, is present in cruciferous vegetables [15]. Accumulating epidemiological evidence indicates an inverse relationship between intake of cruciferous vegetables and the risk of ovarian cancer [16]. Several studies, including those from our laboratory, have suggested that DIM possesses chemopreventive and therapeutic properties [17-19]. Moreover, DIM was shown to be non-toxic to normal cells [20]. A recently concluded DIM clinical trial demonstrated that 50% of cervical cancer patients showed improvement [21]. It is also currently in clinical trials for prostate cancer [22]. The effects of DIM were recently discussed in detail by Banerjee *et al*. [23]. In our previous study, we showed that DIM exhibits antiproliferative properties in ovarian cancer cells by causing G2/M cell cycle arrest [17]. However, the mechanism by which DIM inhibits proliferation of ovarian cancer cells was not clear.

In the present study, we provide evidence that DIM induces apoptosis in ovarian cancer cells by blocking the activation of STAT3 and its downstream effector molecules, while IL-6 treatment or overexpression of STAT3 significantly protects ovarian cancer cells from DIM-induced apoptosis. Our results also show that DIM suppresses angiogenesis and metastasis. In addition, DIM potentiates the effect of cisplatin in inducing apoptosis and inhibiting angiogenesis and metastasis. As a proof of concept, *in vivo *efficacy of DIM alone and in combination with cisplatin also was evaluated.

## Methods

### Chemicals

BR-DIM was a kind gift from Dr Michael Zeligs (Bio Response, Boulder, CO, USA). Cisplatin was obtained from Novaplus (Bedford, OH, USA). Antibodies against cleaved (Cl)-caspase 3, Cl-poly(ADP-ribose) polymerase (PARP), p-STAT3 (Tyr-705), STAT3, Mcl-1, and survivin were obtained from Cell Signaling Technology (Danvers, MA, USA). VEGF antibody was obtained from R&D systems (Minneapolis, MN, USA), Lamin B was from Santa Cruz Biotechnologies (Santa Cruz, CA, USA), and p-STAT3 (Ser-727) and HIF-1α were obtained from Abcam Inc. (Cambridge, MA, USA). Actin antibody, IL-6, MCDB105 and Medium 199 were procured from Sigma Aldrich (St Louis, MO, USA). RPMI and McCoy 5A were purchased from Mediatech (Manassas, VA, USA). NE-PER nuclear fractionation kit was from Thermo Scientific. EZ-TFA transcription factor assay kit was obtained from Upstate (Millipore, Billerica, MA, USA). Dual luciferase kit was bought from Promega (Madison, WI, USA). The VEGF Elisa kit was from Invitrogen (Carlsbad, CA, USA) and FuGENE 6 was obtained from Roche (South San Francisco, CA, USA). IL-6 secretion ELISA kit was from ebiosciences (San Diego, CA, USA).

### Cell cultures

SKOV-3, OVCAR-3 and TOV-21G cells lines were procured from American Type Culture Collection (ATCC; Manassas, VA, USA). SKOV-3 cells were maintained in McCoy's 5A medium supplemented with 10% fetal bovine serum (FBS). OVCAR-3 cells were maintained in RPMI medium supplemented with 20% FBS, 10 mM sodium pyruvate, 10 mM 4-(2-hydroxyethyl)-1-piperazineethanesulfonic acid (HEPES), 10 mg/l bovine insulin and 4.5 g/l glucose. Human normal ovarian surface epithelium cells (NOSE) were a kind gift from Dr Jinsong Liu at MD Anderson, Houston, TX, USA. NOSE cells were previously transfected with the SV40 early region expressing large T and small t antigens as described elsewhere [24]. NOSE and TOV21G cells were maintained in 1:1 mixture of MCDB105 and Medium 199 supplemented with 15% FBS. The A2780 cell line (a kind gift from Dr Thomas Hamilton, Fox Chase Cancer Center, Philadelphia, PA, USA) was maintained in RPMI media supplemented with 10% FBS and 2.7 units/ml insulin. OVCAR-429 and OVCAR-433 (a kind gift from Dr Laurie Hudson, University of New Mexico) were maintained in Dulbecco's modified Eagle medium (DMEM) with 10% FBS. A 1% antibiotic mixture was used in all the above media. All the cell lines were maintained at 37°C in a humidified incubator circulated with 5% CO_2_/95% air. The cell survival assay was performed as described by our group previously [17].

### Annexin V apoptosis assay

SKOV-3 cells were plated at a density of 0.3 × 10^6 ^cells per well in a six-well plate and allowed to attach overnight. Cells were then treated with or without DIM. After 24 h cells were exposed to IL-6 for 15 minutes, washed, suspended in binding buffer, and incubated for 15 minutes with annexin V-FITC (BD Biosciences, San Jose, CA, USA). Fluorescence was measured using a C6 Accuri flow cytometer (Ann Arbor, MI, USA) with a minimum of 10,000 events per sample as previously described by our group [25].

### Western blot analysis

SKOV-3, OVCAR-3, TOV-21G and A2780 cells were exposed to varying concentrations of DIM alone or in combination with cisplatin. Cells were collected, lysed, and about 20 to 80 μg protein was subjected to SDS gel electrophoresis followed by immunoblotting as previously described by our group [26].

### Nuclear fractionation

SKOV-3, OVCAR-3, TOV-21G, or A2780 cells were plated at a density of 1 × 10^6 ^in 100 mm culture dishes and exposed to different concentrations of DIM for 24 h. Nuclear fraction was extracted using NE-PER kit from Thermo Scientific according to the manufacturer's instructions.

### STAT3 DNA binding activity

DNA binding activity of STAT3 was measured by Universal EZ-TFA transcription factor assay colorimetric kit. SKOV-3 or OVCAR-3 cells were treated with or without DIM for 24 h. Nuclear extracts were used to determine the specific STAT3 DNA binding activity as previously described by our group [27].

### STAT3 luciferase reporter assay

Transcriptional activity of STAT3 was determined in SKOV-3 and OVCAR-3 cells by transfecting the cells with 2 μg pLuc-TK/STAT3, which encoded firefly luciferase under the control of STAT3 promoter, and with 0.2 μg of a pRL-TK, which constitutively expressed Renilla luciferase, the latter as a transfection efficiency control. At 24 h after transfection, cells were treated with or without DIM for 24 h. Whole cell lysates were collected using passive lysis buffer provided by dual luciferase reporter assay kit. Renilla and firefly luciferase activities were measured by a luminometer. Firefly luciferase activities were corrected for Renilla values and then normalized relative to dimethylsulfoxide (DMSO) control as previously described [27].

### IL-6 treatment

SKOV-3, OVCAR-3, or TOV-21G cells were treated with 75 μM DIM for 24 h followed by incubation with 10 ng/ml IL-6 for 15 minutes. Cells were then processed for apoptosis assay or western blotting as described above.

### MG132 treatment

Since HIF-1α is proteasomally degraded, SKOV-3 and OVCAR-3 cells were pretreated with 10 μM MG132 for 1 h and then treated with 75 μM DIM for 6 h. In another experiment, after DIM treatment, cells were treated with or without 10 ng/ml IL-6 for 15 minutes. Samples were processed for western blotting as described above.

### STAT3α overexpression

A total of 0.3 × 10^6 ^SKOV-3 cells were plated in McCoy's 5A medium containing 10% FBS without antibiotics and allowed to attach overnight. Complexes were prepared by incubating 2 μg STAT3α plasmid with 6 μl FuGENE 6 transfection reagent in 100 μl McCoy media without serum or antibiotic for 1 h. These complexes were then added to the cells. At 6 h after transfection, the media was replaced by regular media. After 24 h of transfection, cells were further treated with or without DIM for 24 h.

### STAT3 small hairpin (sh)RNA

A total of 0.3 × 10^6 ^SKOV-3 cells were plated in McCoy's 5A medium containing 10% FBS without antibiotics and allowed to attach overnight. Complexes were prepared by incubating 2 μg STAT3 shRNA with 6 μl FuGENE 6 transfection reagent in 100 μl McCoy media without serum or antibiotic for 1 h. These complexes were then added to the cells. At 6 h after transfection, media was replaced by regular media. After 24 h of transfection, cells were processed for apoptosis assay as described above.

### Estimation of IL-6 secretion by ELISA

About 10,000 SKOV-3, OVCAR-3 or OVCAR-429 cells were plated per well in a 96-well plate. Cells were starved overnight followed by treatment with DIM for 24 h. After 24 h, medium was collected and processed for measuring secreted IL-6 levels using ELISA kit according to manufacturer's instructions.

### Aortic ring assay

Aortic ring spouting assay was performed as previously described [28]. In brief, 1 mm long rings were excised from rat thoracic aorta. The rings were submerged in 350 μL Matrigel (BD Biosciences) containing 50 ng/ml IL-6. After a 24-h incubation, DIM, cisplatin, or both were added to the rings and incubated for an additional 3 to 5 days. The aortic rings that formed microvascular-like sprouts were photographed under light microscope (Olympus Inc., PA, USA) and the results were quantified by ImageJ V.1.43 software provided by NIH.

### Estimation of VEGF secretion by ELISA

Secreted VEGF levels in DIM treated SKOV-3 and OVCAR-3 cell culture medium were measured using ELISA kit according to manufacturer's instructions.

### Wound healing assay

Wound healing assay was performed as described previously [29]. Confluent monolayers of SKOV-3; OVCAR-3; and TOV-21G cells in six well plates were scratched with a 1 ml pipette tip and incubated in respective medium containing 50 μM DIM. Cells were photographed under a light microscope (Olympus) at 0, 24 and 48 h and the results were quantified by ImageJ software (NIH).

### Transwell cell invasion assay

Cell invasion was performed according to the manufacturer's instructions in transwell Boyden's chambers with 8.0 μm pore size filters (BD Biosciences). Briefly, cells were serum starved overnight and harvested by trypsinization. A suspension of 20,000 SKOV-3 cells in 600 μL McCoy medium containing 1% serum were seeded on the upper well of the Boyden's chamber and the lower chamber was filled with 1.5 ml of media containing 1% serum. After incubation for 2 h, DIM or cisplatin or both were added to the upper chamber whereas 10% FBS and 20 ng/ml VEGF was added to the lower chamber as chemoattractant. After incubation for 24 h, cells from the upper chamber were removed by wiping with a cotton swab, and the filter was fixed with 10% trichloroacetic acid (TCA) and stained with 0.4% (w/v) sulforhodamine B (SRB) solution. The filters containing stained cells were removed from the transwell chambers and individually transferred to individual wells in a 96-well plate. The SRB dye retained on the filter was extracted with 10 mM Tris buffer and the absorbance was measured at 570 nm using a microplate reader (BioTek Instruments, VT, USA). Assays were performed in duplicates and data was expressed as percent migration with control.

### *In vivo *xenograft experiment

Female athymic nude mice, 4 to 6 weeks old, were purchased from Charles River Laboratories (Wilmington, MA, USA). The use of mice and their treatment was approved by Institutional Animal Care and Use Committee (IACUC), Texas Tech University Health Sciences Center, and all the experiments were carried out in strict compliance with regulations. Mice were fed with antioxidant-free AIN-76A special diet for a week before starting the experiment. About 5 × 10^6 ^SKOV-3 cells were injected subcutaneously into both right and left flanks. Eight mice were assigned randomly to each group. Since each mouse was implanted two xenografts, each group had 16 tumors. Once each mouse achieved a tumor of about 90 mm^3^, the control group received PBS whereas mice in the treatment group received 3 mg DIM suspended in PBS by oral gavage every day. At day 34, 5 mg/kg cisplatin was injected intraperitoneally to the treatment group mice. Beginning on the 7th day after cell implantation, tumor volume was measured three times a week using vernier calipers until day 48, as previously described by our group [27]. On day 48 the mice were killed and tumors were removed for western blot analysis.

### SRB cell survival assay

Around 5,000 cells in 0.1 ml medium were plated per well in 96 well plates and allowed to attach overnight. Desired concentrations of DIM were added to the cells and incubated at 37°C for 24 h. The cells were then processed and stained with 0.4% SRB solution and the absorbance was read at 570 nm using a Biotek plate reader as described by our group previously [17].

### Statistical analysis

All the statistical analyses were performed using Prism 5.0 (GraphPad Software Inc., San Diego, CA, USA). The data represents mean values with SD. The Student's t test was used to compare the control and treated groups. In experiments involving more than three groups, non-parametric analysis of variance followed by Bonferroni *post hoc *multiple comparison test was used. All statistical tests were two sided. Differences were considered statistically significant when the *P *value was less than 0.05.

## Results

### DIM induces apoptosis in ovarian cancer cells

**Figure 1:**
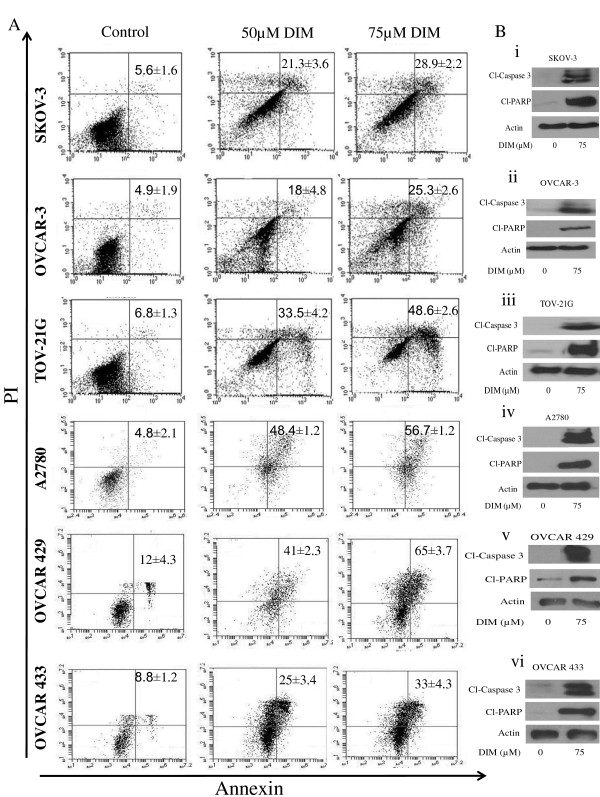
**Diindolylmethane (DIM) induces apoptosis in ovarian cancer cells**. **(A) **SKOV-3, OVCAR-3, TOV-21G, A2780, OVCAR-429 and OVCAR-433 cells were treated with or without 50 μM or 75 μM DIM for 24 h. Cells that were positive for annexin or propidium iodide (PI) or both were measured using flow cytometry. Representative images are shown. **(B) **Representative western blots of cleaved caspase 3 and cleaved poly(ADP-ribose) polymerase (PARP) from the lysates collected from **(i) **SKOV-3, **(ii) **OVCAR-3, **(iii) **TOV-21G, **(iv) **A2780, **(v) **OVCAR-429 and **(vi) **OVCAR-433 cells treated with or without 75 μM DIM.

We previously reported that DIM inhibits the growth of ovarian cancer cells by causing cell cycle arrest [17]; however, the exact mechanism was not clear. The apoptosis-inducing effect of DIM was determined in ovarian cancer cells using annexin V-FITC. Treatment of SKOV-3 cells with 50 μM or 75 μM DIM for 24 h resulted in an approximately 2.8-fold to 5.2-fold increase in apoptosis (Figure 1A). To rule out the cell specific effects of DIM, we evaluated DIM-induced apoptosis in five other ovarian cancer cell lines. Our results showed that apoptosis induced by DIM was nearly 3.6-fold to 5.2-fold higher in OVCAR-3, 4.9-fold to 7.1-fold higher in TOV-21G, 10.1-fold to 11.8-fold higher in A2780, fourfold to fivefold higher in OVCAR-429 and threefold to fourfold higher in OVCAR-433 cells (Figure 1A). Apoptosis-inducing effects of DIM were further confirmed by western blot analysis that revealed the cleavage of caspase 3 and PARP in DIM-treated ovarian cancer cells (Figure 1B). These results clearly establish that DIM induces apoptosis in ovarian cancer cells.

### DIM targets the STAT3 pathway

**Figure 2:**
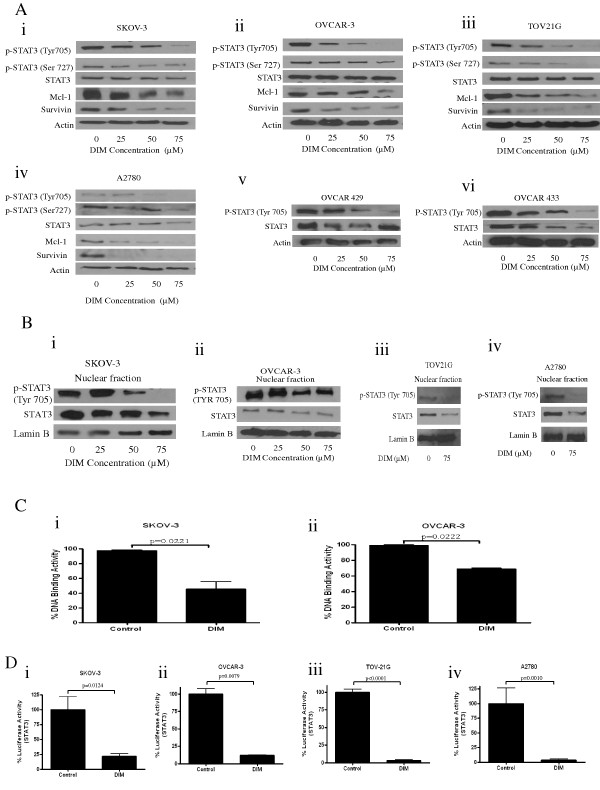
**Diindolylmethane (DIM) inhibits signal transducer and activator of transcription 3 (STAT3) pathway in ovarian cancer cells. (A) **Representative blots showing the concentration-dependent effect of DIM on phosphorylated (p)-STAT3 (Tyr-705), p-STAT3 (Ser-727), STAT3, Mcl-1, survivin in **(i) **SKOV-3, **(ii) **OVCAR-3, **(iii) **TOV-21G and **(iv) **A2780 ovarian cancer cells. Concentration-dependent effect of DIM on p-STAT3 (Tyr-705) and STAT3 in **(v) **OVCAR-429 and **(vi) **OVCAR-433 cells. Actin was used as loading control. **(B) **Effect of DIM on nuclear translocation of p-STAT3 and STAT3. Representative blots of nuclear lysates of **(i) **SKOV-3, **(ii) **OVCAR-3, **(iii) **TOV21G and **(iv) **A2780 cells treated with DIM. Lamin B was used as loading control. **(C) **Effect of DIM on STAT3 DNA binding. **(i) **SKOV-3 cells or **(ii) **OVCAR-3 cells were treated for 24 h with or without 75 μM DIM and nuclear cell extracts were tested for STAT3 DNA-binding activity as measured by the Universal EZ-TFA transcription factor colorimetric assay. **(D) **Effect of DIM on STAT3-regulated luciferase reporter activity. STAT3 luciferase transcriptional activity was determined in **(i) **SKOV-3, **(ii) **OVCAR-3, **(iii) **TOV21G or **(iv) **A2780 cells. Firefly luciferase activities were corrected for Renilla luciferase levels and then normalized relative to the control, which was considered as 100%. Means and SD of two independent experiments performed in triplicate are shown. The Student's t test was used for statistical analysis to compare control and DIM treatment.

Our next step was to investigate the mechanism by which DIM induces apoptosis. STAT3 is activated in almost 90% of ovarian cancers. Overexpression of STAT3 was reported in stage III and IV ovarian tumors [14]. We hypothesized that DIM induces apoptosis in ovarian cancer cells by inhibiting STAT3, and then systematically tested our hypothesis. SKOV-3, OVCAR-3, TOV-21G, A2780, OVCAR-429 and OVCAR-433 cells treated with varying concentrations of DIM were subjected to western blotting. Our results clearly show that DIM substantially inhibits the activation of STAT3 by suppressing phosphorylation at Tyr-705 and Ser-727 (Figure 2A i-vi). The protein levels of STAT3 decreased modestly with DIM treatment. Our results further show that the expression of Mcl-1 and survivin were drastically decreased by DIM treatment in a concentration-dependent manner in all four cell lines (Figure 2A i-iv). Regulated by STAT3, both Mcl-1 and survivin have been implicated in cancer growth. These results demonstrate that DIM targets STAT3 pathway in ovarian cancer cells.

### DIM inhibits nuclear translocation of STAT3

Once phosphorylated at Tyr-705, STAT3 forms a homodimer and translocates into the nucleus where it gets involved in the transcription of cell survival genes such as Mcl-1, survivin. Our results show that nuclear translocation of STAT3 was blocked by DIM treatment in SKOV-3, OVCAR-3, TOV-21G and A2780 cells (Figure 2B i-iv) and strengthen our hypothesis that DIM inhibits the growth of ovarian cancer cells by blocking STAT3.

### DIM inhibits DNA binding activity and transcriptional activity of STAT3

Upon translocating to the nucleus, STAT3 binds to specific response elements in the promoter regions of its responsive genes. Since we observed that DIM blocked the nuclear translocation of STAT3, our next step was to determine whether DIM can inhibit DNA binding and transcriptional activity of STAT3. DNA-binding activity of STAT3 was determined by Universal EZ-TFA transcription factor colorimetric assay using the nuclear lysates of cells treated with or without DIM. Our results show that DIM treatment resulted in approximately 60% inhibition of DNA binding activity in SKOV-3 cells (Figure 2C i). Around 30% inhibition of DNA binding activity was observed in OVCAR-3 cells by DIM (Figure 2C ii).

We then evaluated the transcriptional activity of STAT3 by luciferase assay. We observed a dramatic reduction in STAT3 transcriptional activity by DIM treatment. In SKOV-3 and OVCAR-3 cells, transcriptional activity was reduced by 80% (Figure 2D i, ii). Similarly, in TOV21G and A2780 cells, transcriptional activity decreased by 90% (Figure 2D iii, iv).

### DIM inhibits IL-6-induced activation of STAT3

**Figure 3:**
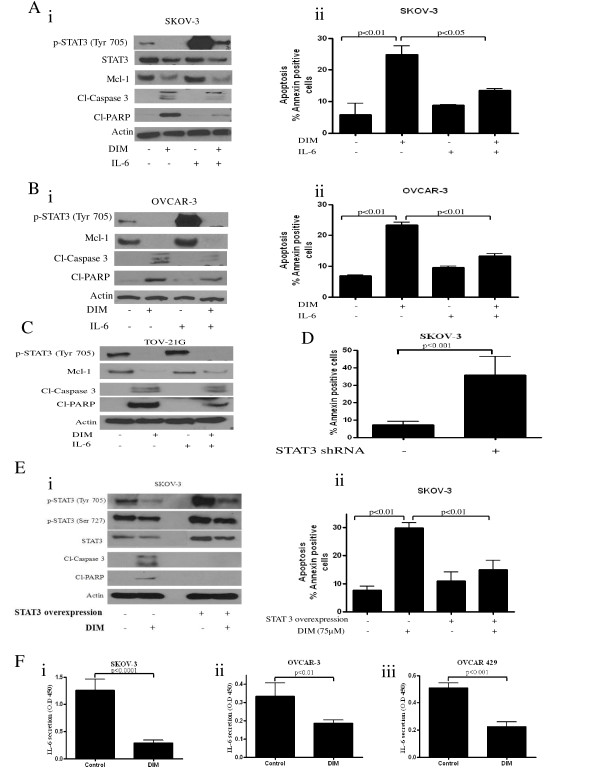
**Diindolylmethane (DIM) suppresses signal transducer and activator of transcription 3 (STAT3) activated by interleukin (IL)-6 or in cells overexpressing STAT3**. Effect of DIM on STAT3 activation and apoptosis. **(A) (i) **SKOV-3, **(B) (i) **OVCAR-3 or **(C) (i) **TOV-21G cells were stimulated with 10 ng/ml IL-6 for 15 minutes after treatment with 75 μM DIM for 24 h. Whole cell lysates were resolved on 10% SDS-PAGE for the analysis of STAT3 phosphorylation at Tyr-705 and Ser-727, STAT3 expression, and cleavage of caspase 3 and poly(ADP-ribose) polymerase (PARP). Actin was used as a control for loading. Effect of IL-6 on apoptosis was also determined in (A) **(ii) **SKOV-3 and (B) **(ii) **OVCAR-3 cells. **(D) **Effect of STAT3 small hairpin (sh)RNA on apoptosis was also determined in SKOV-3 cells. **(E) **Effect of DIM on STAT3 activation, STAT3 expression, and apoptosis in SKOV-3 cells overexpressing STAT3. Cells were transfected with an empty vector or a plasmid expressing STAT3 for 24 h and treated with 75 μM DIM for another 24 h. (E) **(i) **Whole cell lysates were analyzed by western blotting. (E) **(ii) **Effect of STAT3 overexpression on DIM-induced apoptosis was determined by flow cytometry. **(F) **Effect of DIM on IL-6 secretion was determined in (F) **(i) **SKOV-3, **(ii) **OVCAR-3 and **(iii) **OVCAR 429 cells. The experiments were repeated three times and similar results were obtained. The differences between all the groups were compared by non-parametric analysis of variance with Bonferroni *post hoc *comparisons.

STAT3 can be activated by IL-6, a cytokine that binds to its receptor and phosphorylates at Tyr-705. Exposing SKOV-3 or OVCAR-3 cells to 10 ng/ml IL-6 for 15 minutes led to an enormous activation of STAT3 (Figure 3A,B). However, IL-6 treatment failed to induce a significant activation of STAT3 in DIM treated cells (Figure 3A i, B i, C). STAT3 protein level was not affected by IL-6 treatment (Figure 3A i). We further observed that IL-6 treatment significantly blocked DIM-induced cleavage of caspase 3 and PARP (Figure 3A). These results were confirmed by annexin staining showing that IL-6 significantly blocked DIM-induced apoptosis (Figure 3A ii). Similarly, in OVCAR-3 cells, apoptosis induced by DIM was significantly blocked by IL-6 (Figure 3B ii).

### Ectopic expression of STAT3 abrogates DIM-induced apoptosis

To further strengthen our observation that DIM-induced apoptosis was mediated by STAT3 inhibition in ovarian cancer cells, we transiently transfected SKOV-3 cells with STAT3 encoding plasmid for 24 h, resulting in an almost twofold increase in the expression of STAT3 (Figure 3E i). Correspondingly, phosphorylation of STAT3 at both Tyr-705 and Ser-727 also increased substantially. STAT3 overexpression completely protected SKOV-3 cells from DIM-induced apoptosis as evaluated by cleavage of caspase 3 and PARP (Figure 3E i). These observations were further confirmed by annexin V apoptosis assay by flow cytometry (Figure 3E ii). Taken together, our results clearly indicate that apoptosis induced by DIM was almost completely blocked in the cells overexpressing STAT3 (Figure 3D ii), establishing STAT3 as a target of DIM in ovarian cancer cells.

### Silencing STAT3 using shRNA induces apoptosis in SKOV-3 cells

To address whether or not inhibition of STAT3 induces apoptosis in ovarian cancer cells, we silenced STAT3 using shRNA in SKOV-3 cells. Supporting our hypothesis, our results show that silencing STAT3 resulted in about 35% apoptosis in SKOV-3 cells suggesting the critical role of STAT3 in ovarian cancer cells (Figure 3D). Our results are in agreement with a previously published study that showed that STAT3 specific inhibitor (AG490) induced apoptosis in ovarian cancer cells [30].

### DIM inhibits IL-6 secretion

IL-6, a cytokine, activates STAT3 by phosphorylating it at Tyr-705. High levels of circulating IL-6 were observed in ovarian cancer patients [31]. Since we observed that DIM can suppress IL-6-induced activation of STAT3 in ovarian cancer cells, we hypothesized that DIM mediated inhibition of STAT3 is in fact by inhibition of IL-6. We therefore measured IL-6 secretion by ovarian cancer cells using a commercial ELISA kit. Our results demonstrate that DIM drastically blocks the secretion of IL-6 in SKOV-3, OVCAR-3 and OVCAR-429 cells (Figure 3F i-iii). Reduction in IL-6 secretion levels ranged from 60% to 80% in three different cell lines.

### DIM inhibits cell migration and neovascularization

**Figure 4:**
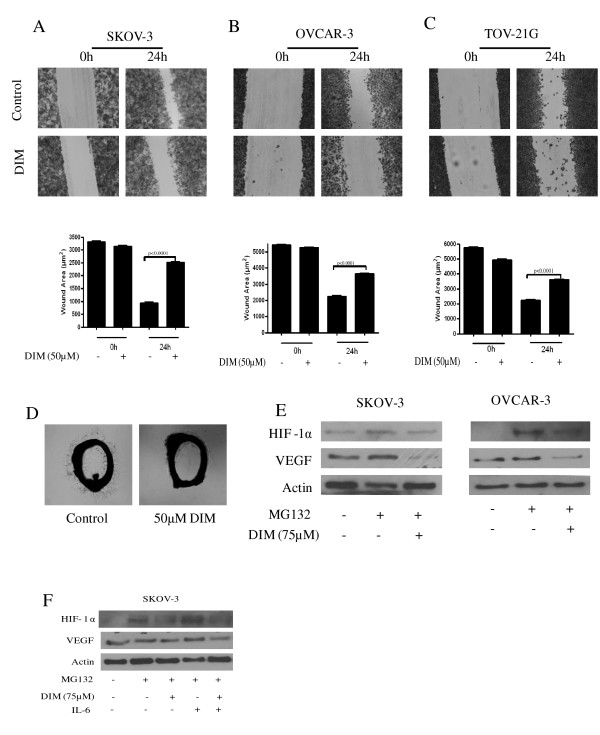
**Diindolylmethane (DIM) blocks cell migration and neovascularization by inhibiting hypoxia-inducible factor 1α (HIF-1α) and vascular epithelial growth factor (VEGF). (A) **SKOV-3, **(B) **OVCAR-3 or **(C) **TOV21G cells were plated, scratched with a pipette tip and incubated in the absence or presence of 50 μM DIM. Photographs were taken at 0 h and 24 h using an inverted microscope. The wound area in DIM treated and control cells were quantified by ImageJ software. Results are presented as means ± SD of triplicates. (D) DIM inhibits interleukin (IL)-6-induced vessel sprouting *ex vivo*. Representative photographs are presented. **(E) **Effect of DIM on proangiogenic proteins was analyzed by western blotting. SKOV-3 or OVCAR-3 cells were treated with 10 μM MG132 for 1 h and then exposed to 75 μM DIM for 6 h. **(F) **Cells were treated as above and then treated with IL-6 for 15 minutes. Representative blots of HIF-1α and VEGF are shown. Blots were further stripped and probed with actin. Experiments were performed independently three times. The Student's t test was used for statistical analysis to compare control and DIM treatment.

Recent literature suggested a novel role of STAT3 in angiogenesis and metastasis. Since DIM suppressed the activation of STAT3 in ovarian cancer cells, we wanted to test whether DIM can inhibit invasion and angiogenesis. We first determined the anti-invasive potential of DIM by evaluating cell migration in a wound healing assay. A wound was made on confluent monolayer cells with a pipette tip and, after washing with fresh medium, cells were treated with or without 50 μM DIM. Our results revealed that after 24 h DIM treatment, SKOV-3 cells migrated into 20% of the wounded area, whereas control cells migrated into 80% of the wounded area, showing the potential anti-invasive property of DIM (Figure 4A). Similar observations were made in OVCAR-3 and TOV-21G cells (Figure 4B, C).

Since several studies have established the role of activated STAT3 in tumor angiogenesis, we next sought to determine the antiangiogenic potential of DIM. We used an aortic ring model where microvessel sprouting occurs in response to growth stimulatory signals. Treatment with IL-6 induced a massive microvessel formation on rat aortic rings after 24 h of incubation. However, microvessel sprouting was drastically reduced in aortic rings treated with DIM (Figure 4D).

STAT3 positively regulates HIF-1α and VEGF under hypoxia. VEGF has also been shown to be regulated by STAT3 in an HIF-1α-dependent manner [13]. Under normal conditions, HIF-1α is constitutively synthesized and subjected to proteasomal degradation to maintain minimal levels. In order to determine the effect of DIM on HIF-1α expression, cells were treated with MG132, a known proteasomal inhibitor, in the presence or absence of DIM. Our results show that HIF-1α expression was drastically increased after 6 h of MG132 treatment. However, treatment of SKOV-3 or OVCAR-3 cells with DIM after MG132 treatment did not result in the accumulation of HIF-1α (Figure 4E). These results indicate that DIM suppresses HIF-1α induction (Figure 4E). To delineate the role of STAT3 in DIM-mediated HIF-1α down regulation, cells were treated with IL-6, which activates STAT3 phosphorylation, leading to the induction of HIF-1α. Our results further show that IL-6-induced HIF-1α expression was significantly attenuated by DIM (Figure 4F). VEGF expression, which normally is regulated by HIF-1α, was also substantially reduced by DIM treatment (Figure 4F). These results provide critical evidence that DIM inhibits angiogenesis in ovarian cancer cells by downregulating HIF-1α and VEGF through STAT3.

### DIM potentiates the effect of cisplatin

**Figure 5:**
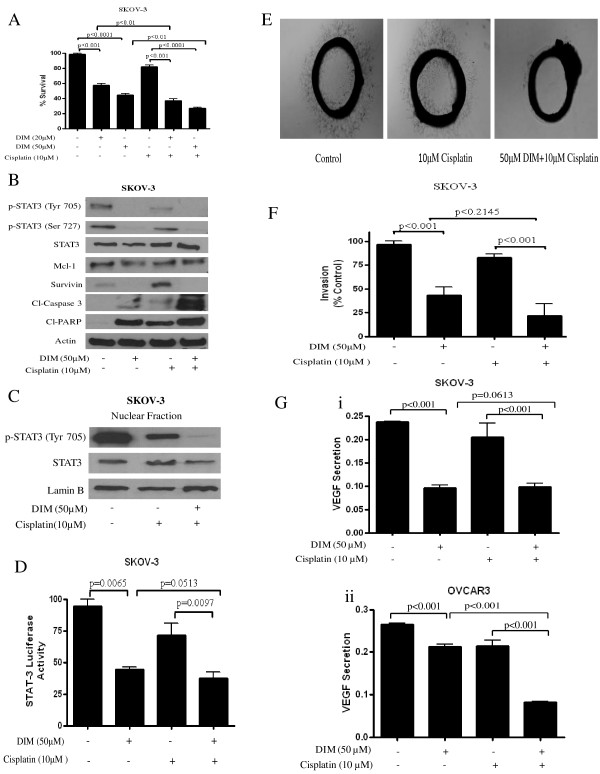
**Diindolylmethane (DIM) potentiates the effect of cisplatin in ovarian cancer cells. (A) **SKOV-3 cells were exposed to 20 or 50 μM DIM for 24 h, followed by exposure to 10 μM cisplatin for another 24 h. Survival of control and treated cells was evaluated by sulforhodamine B assay. **(B) **Combination effect of cisplatin and DIM on the signal transducer and activator of transcription 3 (STAT3) pathway. Representative blots of control and treated cells were examined for Tyr-705 and Ser-727 STAT3, STAT3, Mcl-1, survivin, cleaved poly(ADP-ribose) polymerase (PARP), and cleaved caspase 3. Blots were stripped and probed with actin. **(C) **Effect of combination treatment on nuclear localization of STAT3. Nuclear fractions from cells treated with or without 50 μM DIM or 10 μM cisplatin or both were subjected to western blotting. Representative blots of Tyr-705 STAT3 and STAT3 are shown. Lamin B was used as loading control. **(D) **Luciferase activity in combination treatment was measured in SKOV-3. Whole cell lysates were collected and firefly luciferase activities were corrected for Renilla luciferase levels and then normalized relative to the control, which was considered as 100%. **(E) **Aortic rings (1 mm) were harvested from Sprague-Dawley rats, immersed in matrigel, and treated with interleukin (IL)-6 (50 ng/ml) in the absence or presence of cisplatin (10 μM) with or without DIM (50 μM) for 4 days, and then photographed under a microscope (4 ×). Representative photographs are shown. **(F) **DIM inhibits the invasion of SKOV-3 cells. Invasion assay was performed using Boyden's chamber (BD Sciences) according to manufacturer's instructions. **(G) **DIM inhibits vascular epithelial growth factor (VEGF) secretion. Cells were plated, stimulated with VEGF, and treated with DIM for 24 h. Media was collected and assayed for VEGF by ELISA (Invitrogen) kit according to manufacturer's instructions. All experiments were performed independently three times. The differences between all the groups were compared by non-parametric analysis of variance with Bonferroni *post hoc *comparisons.

We wanted to determine whether DIM can potentiate the effect of cisplatin, a drug used to treat ovarian cancer patients. Cisplatin usually is associated with several side effects such as renal toxicity and reduction of white and red blood cells. Moreover, tumors often become resistant to cisplatin therapy. Hence, enhancing the effect of cisplatin at low doses by agents that are non-toxic may be a better strategy, not only for effective treatment but also to reduce harmful side effects. To test whether or not DIM potentiates the effect of cisplatin, SKOV-3 cells were treated with 20 μM or 50 μM DIM for 24 h followed by exposure to 10 μM cisplatin for an additional 24 h. It is important to mention that the IC_50 _of cisplatin in SKOV-3 cells is 40 μM; we used one-quarter of the IC_50 _concentration. Growth inhibition in combination treatment was significantly higher as compared to either treatment alone (Figure 5A). We observed around 50% to 70% reduction in the survival of SKOV-3 cells treated with a combination of DIM and cisplatin as compared to 28% by cisplatin alone (*P *< 0.001). We calculated the combination index (CI) for DIM and cisplatin combination treatment in SKOV-3 cells. CI values for the combination of cisplatin with 20 μM and 50 μM DIM treatment were 0.45 and 0.56, respectively, indicating the synergistic effect of DIM with cisplatin.

We next wanted to identify the mechanism behind the synergistic effects of DIM with cisplatin. We hypothesized that the enhanced effect of cisplatin by DIM was due to modulation of STAT3 pathway. In agreement with our hypothesis, combination treatment strongly suppressed the activation of STAT3 at Tyr-705 or Ser-727 as compared to cisplatin only treatment (Figure 5B). Mcl-1 also was downregulated in a similar manner. It is important to note that survivin, which is a positive regulator of growth and a major downstream target of STAT3, was drastically upregulated by cisplatin treatment alone. Nonetheless, DIM completely blocked cisplatin-mediated upregulation of survivin in the combination treatment (Figure 5B). Increased cleavage of caspase 3 and PARP by combination treatment indicated significantly more apoptosis as compared to either treatment alone. Furthermore, combination treatment completely abolished the phosphorylation and reduced the expression of STAT3 in the nuclear fraction of cells (Figure 5C). Similarly, transcriptional activity of STAT3 was also significantly suppressed by combination treatment (Figure 5D).

We then wanted to know if combination treatment could inhibit angiogenesis in a rat aorta model. Our data showed that after 72 h, microvessel sprouting in rat aorta was not suppressed by cisplatin treatment (Figure 5E). However, combination treatment completely blocked microvessel sprouting (Figure 5E). The combination treatment of DIM with cisplatin also significantly blocked the invasion of the cells as evaluated by the Boyden's chamber technique (Figure 5F). However, cisplatin alone had hardly any inhibitory effect on the invasion of cells (Figure 5F). We also tested whether DIM alone or in combination inhibited VEGF secretion. Our results clearly demonstrate that DIM treatment in combination with cisplatin decreased the VEGF secretion by 60% as compared to control or cisplatin treatment only in both SKOV-3 and OVCAR-3 cells (Figure 5G).

### DIM treatment alone and with cisplatin inhibits ovarian tumor growth

**Figure 6:**
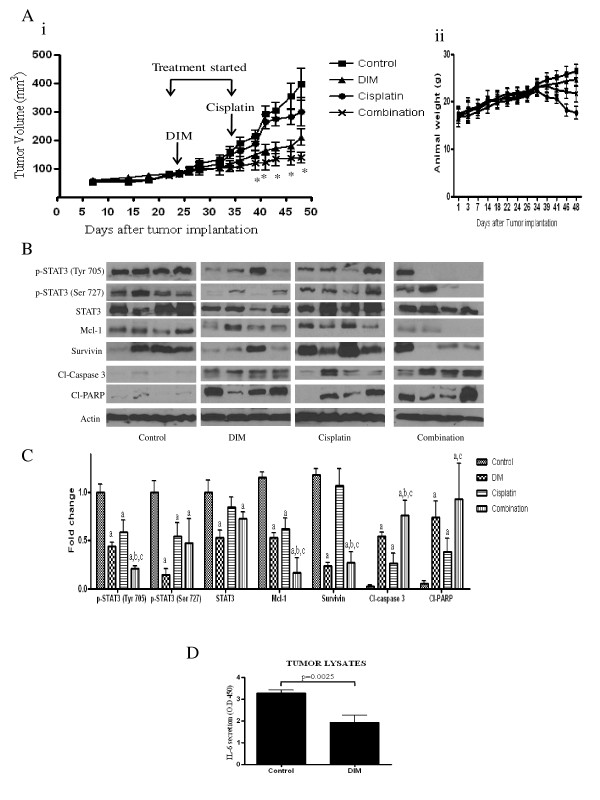
**Diindolylmethane (DIM) suppresses the growth of ovarian tumors alone and in combination with cisplatin by inhibiting signal transducer and activator of transcription 3 (STAT3) in nude mice**. SKOV-3 tumor cells were implanted into athymic nude mice. Once each mouse had a palpable tumor, mice received 3 mg/day DIM by oral gavage every day or 5 mg/kg cisplatin intraperitoneally twice a week or both. **(A) (i) **Effect of DIM on tumor growth. *P < 0.05 when compared to control. **(ii) **Tumor weight of mice from different groups during the course of *in vivo *study. **(B) **Inhibition of STAT3 signaling in the tumors of mice administered with DIM alone or in combination with cisplatin. Tumors from control and treated mice were excised on day 48 after implantation, lysed and analyzed by western blotting for Tyr-705 STAT3, Ser-727 STAT3, total STAT3, Mcl-1, survivin, cleaved poly(ADP-ribose) polymerase (PARP) and cleaved caspase 3. Blots were stripped and reprobed with actin antibody to verify equal protein loading. Each lane represents a different tumor sample. **(C) **Densitometric quantitation of western blotting represented above. Legends on the bars indicate (a) statistically significant compared to control, (b) statistically significant compared to DIM, (c) statistically significant compared to cisplatin. **(D) **Levels of interleukin (IL)-6 in tumors from control mice and DIM treated mice. The differences between all the groups were compared by non-parametric analysis of variance with Bonferroni *post hoc *comparisons.

We demonstrated that DIM induces apoptosis in ovarian cancer cells and enhances the effect of cisplatin by inhibiting STAT3 pathway in culture models. To validate these effects *in vivo*, we performed a tumor xenograft assay. About 5 × 10^6 ^SKOV-3 cells were injected subcutaneously into both the right and left flanks of female athymic nude mice. Once each mouse had a tumor of about 90 mm^3^, they were randomized into four groups. DIM treatment started 24 days after tumor implantation, and cisplatin treatment began 10 days later. Our results demonstrated that DIM alone and in combination treatment substantially retarded the growth of SKOV-3 tumors as compared to control or cisplatin treatment. For example, at day 48 the average tumor volume in control mice and cisplatin-treated mice was around 400 mm^3 ^and 300 mm^3^, respectively, whereas the average tumor volume in mice that received DIM alone or in combination with cisplatin was 210 mm^3 ^and 138 mm^3^, respectively (Figure 6A). The combination treatment suppressed tumors by 65% as compared to controls. Interestingly, there was no significant change in the weight of mice treated with DIM as compared to mice in the control group. The weight of mice declined significantly in response to cisplatin treatment. However, the decline in weight in the combination treatment group was not as significant as in the mice receiving cisplatin treatment alone (Figure 6A ii). To determine whether DIM-mediated tumor growth suppression was due to inhibition of STAT3, tumor lysates were subjected to western blotting. As shown in Figure 6B, p-STAT3 (Tyr-705), STAT3, and Mcl-1 were downregulated in the tumors of mice treated with DIM alone or in combination with cisplatin as compared to control or cisplatin treatment. Furthermore, cleavage of caspase 3 and PARP increased significantly in the tumors of mice treated with combination, yet again confirming that DIM suppressed tumor growth by inducing apoptosis *in vivo*. Furthermore, IL-6 levels in tumor lysates from DIM treated mice were significantly less as compared to levels in the tumor lysates from control mice (Figure 6D). Taken together, our results clearly establish that DIM induces apoptosis in ovarian cancer cells *in vitro *and *in vivo *by targeting the STAT3 pathway and synergistically enhancing the effects of cisplatin.

### DIM is minimally toxic to normal human ovarian surface epithelial cells

**Figure 7:**
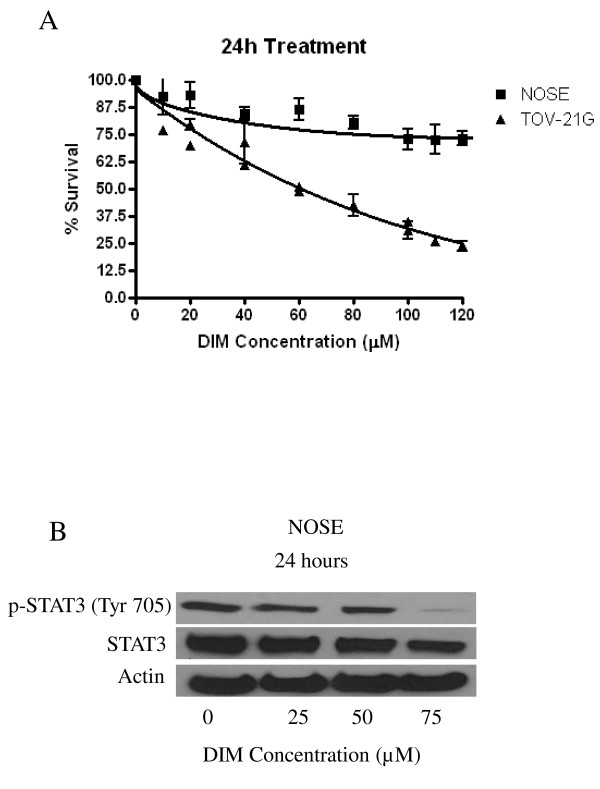
**Diindolylmethane (DIM) is minimally toxic to normal ovarian surface epithelium cells**. **(A) **Effect of varying concentrations of DIM in NOSE and TOV-21G cells was determined by sulforhodamine B cell survival assay. Values are means ± SD of two independent experiments with eight replicates. **(B) **In another experiment, NOSE cells were exposed to different concentrations of DIM for 24 h. Cells were lysed and western blotting was performed as outlined in Methods. Representative immunoblots show the effect of DIM on the expression of phosphorylated signal transducer and activator of transcription 3 (p-STAT3) (Tyr 705) and STAT3. Blots were stripped and probed with actin to ensure proper loading.

In order to determine whether DIM is toxic to normal ovarian cells, we treated NOSE cells with various concentrations of DIM for 24 h. Interestingly, our results showed that DIM treatment caused minimal reduction in the survival of non-tumorigenic ovarian cells (Figure 7A). At a concentration as high as 120 μM, DIM reduced the survival of NOSE cells by about 25%, whereas a 75% reduction in survival was observed in TOV-21G ovarian cancer cells (Figure 7A). These results indicate that DIM is minimally toxic to normal ovarian surface epithelial cells.

To gain further insight into the effect of DIM on p-STAT3, NOSE cells were treated with varying concentrations of DIM for 24 h. Surprisingly, we observed phosphorylation of STAT3 in these cells and 75 μM DIM significantly reduced STAT3 phosphorylation (Figure 7B). Although STAT3 is mainly known to be activated and/or overexpressed in tumorigenic cells, a few recent studies have suggested that IL-6 and STAT3 are activated at low levels in normal ovaries [14,32]. However, the exact mechanism and role of activated STAT3 in normal ovaries is not clear. Our results clearly show that STAT3 is a target of DIM.

## Discussion

Our results demonstrate that DIM suppresses the growth of ovarian cancer cells and potentiates the effect of cisplatin *in vitro *and *in vivo *by targeting STAT3 signaling without being toxic to normal ovarian cells. To the best of our knowledge, this is the first report demonstrating STAT3 as a target of DIM. Accumulated evidence indicates the involvement of STAT3 in the transformation of normal cells into malignant ones [30,33]. STAT3 is overexpressed in ovarian tumors and is associated with ovarian tumorigenesis [14]. A recent study demonstrated that STAT3 is activated in 94% of ovarian cancer patients [14]. Furthermore, STAT3 is also implicated in resistance to chemotherapy in ovarian cancer [5]. Our studies showed that DIM-induced apoptosis in various ovarian cancer cells was mediated by substantially suppressing the phosphorylation of STAT3 at Tyr-705 and Ser-727. Tyrosine phosphorylation is mainly associated with oncogenic status of STAT3. The effects of DIM were not specific to only SKOV-3 cells as similar observations were made in various other ovarian cancer cell lines such as OVCAR-3, TOV-21G, A2780, OVCAR-429 and OVCAR-433. These studies agree with previous reports that demonstrate that inhibiting constitutive activation of STAT3 by STAT3 inhibitor AG490 inhibits tumor growth [34].

Unphosphorylated STAT3 resides in the cytoplasm and is activated by phosphorylation. Once phosphorylated at Tyr-705, STAT3 dimerizes and translocates into the nucleus [35,36]. Around 86% of ovarian tumor tissues have activated STAT3 in the nucleus and not in the cytoplasm [14]. Our results clearly show that the nuclear translocation and activation of STAT3 was substantially reduced by DIM treatment. Inhibition of nuclear translocation in turn inhibits DNA binding activity and transcriptional activity of STAT3 [37,38]. Specific DNA binding of STAT3 leads to transcriptional activation of several downstream molecules such as survivin and Mcl-1. These results demonstrate that DIM treatment blocks the transcriptional and DNA binding activity of STAT3 and downregulates the expression of survivin and Mcl-1. Our observations confirm previous studies indicating that STAT3 inhibition downregulates survivin or Mcl-1 in various cancers [27,39,40]. It is noteworthy that a gene expression profiling of a previous study showed that survivin is a target of DIM [41]. Survivin overexpression is implicated in resistance to cisplatin-induced apoptosis [42,43]. Our results showed that survivin expression was substantially upregulated by cisplatin treatment in ovarian cancer cells. However, DIM treatment completely abolished the overexpression of survivin by cisplatin, suggesting a potential role of DIM in reducing cisplatin-mediated resistance.

Various cytokines and growth factors can activate STAT3. IL-6 is well known to activate STAT3 by phosphorylation at Tyr-705. DIM not only eliminated constitutive activation of STAT3, but also blocked IL-6 mediated activation of STAT3. In addition, to convincingly establish STAT3 as a target of DIM, cells were transfected with STAT3 expression plasmid to activate STAT3 in ovarian cancer cells. Induction of apoptosis by DIM was almost completely blocked in cells overexpressing IL-6 or STAT3, demonstrating that induction of apoptosis in our model was mediated through STAT3 downregulation. Our data indicating that DIM induces apoptosis by inhibiting STAT3 was also supported by our STAT3 knockout studies, which demonstrated the induction of apoptosis when STAT3 was knocked out by shRNA. These results agree with a previous study showing the role of STAT3 in suppressing apoptosis in pancreatic cancer cells [27]. Furthermore, DIM treatment dramatically reduced the secretion of IL-6 not only in ovarian cancer cells but also in tumors. Interestingly, a recent study reported higher IL-6 levels in ovarian cancer patients [31]. This study indicated the IL-6-STAT3-HIF axis as an important target for therapy in ovarian cancer [31]. Our results show that DIM treatment alone or in combination with cisplatin inhibited neovascularization induced by IL-6, suggesting the antiangiogenic potential of DIM. HIF-1α and VEGF play an important role in angiogenesis. HIF-1α is short lived and degrades proteasomally; however, MG132 blocks the degradation of HIF-1α. Our results show that HIF-1α expression retained by MG132 was suppressed as early as 6 h after DIM treatment. IL-6 also activated HIF-1α, which clearly indicates that HIF-1α perhaps is regulated through STAT3 in ovarian cancer cells. It is obvious that inhibition of HIF-1α by DIM was mediated through STAT3 in our model [13,44]. VEGF is another important regulator of angiogenesis and its expression in cancer cells has been shown to correlate with activation of STAT3 [33]. Previous reports have suggested that overexpression of STAT3 increases VEGF expression, leading to angiogenesis [33]. Hence disrupting the activation of STAT3 would inhibit VEGF, blocking angiogenesis [45]. Our results demonstrated that DIM inhibits both VEGF expression and VEGF secretion. This explains the mechanism of the antiangiogenic effects of DIM in ovarian cancer cells in our study.

Cisplatin is a frontline drug used in the treatment of advanced ovarian cancer [46]. Nonetheless, only 15% of patients treated with cisplatin achieve long-term survival; the rest experience persistent or recurrent disease [47]. Moreover, cisplatin is associated with cytotoxicity and resistance to chemotherapy [48,49]. STAT3 activation or overexpression is associated with cisplatin resistance [5]. Our results demonstrate that DIM potentiates the effect of cisplatin at one-quarter of its IC_50 _concentration by inhibiting the activation of STAT3. Combination treatment blocked phosphorylation of STAT3 at both Tyr-705 and Ser-727 when compared to cisplatin treatment alone. Likewise, VEGF secretion and angiogenic sprouting were noticeably reduced in combination treatment as compared to cisplatin alone. These results agree with previous studies that demonstrated inhibition of STAT3 by an analog of aspirin potentiated the effects of cisplatin [50]. The CI provides qualitative information about the nature of drug interaction. CI values less than 1, equal to 1 or greater than 1 indicate synergistic, additive or antagonistic effects, respectively. Our CI values for the combination treatment at both the concentrations of DIM with cisplatin were less than 1, showing the synergistic effects of DIM with cisplatin.

Oral administration of 3 mg DIM per day substantially suppressed the growth of established ovarian tumors, indicating the tumor regression potential of DIM. However, complete regression of tumors was not observed with DIM. When given with cisplatin treatment, DIM further retarded the growth of tumors. The tumors from DIM or DIM plus cisplatin-treated mice clearly demonstrated inhibition of STAT3 signaling and increased apoptosis. Similar to our *in vitro *observations, cisplatin treatment caused a drastic increase in the expression of survivin in the tumors, which was completely suppressed by DIM in combination treatment. Interestingly, mice that received DIM did not show any significant change in body weight when compared with the weight of control mice. However, the mice that received cisplatin and DIM showed a decrease in weight, though this was not as drastic as in the mice that received only cisplatin, suggesting that DIM may reduce the systemic toxicity associated with cisplatin. DIM is a major indole compound present in cruciferous vegetables, and is consumed on a daily basis [51]. A recent single dose phase I clinical trial suggested that 200 mg DIM is well tolerated not only by healthy volunteers but also in patients with cervical cancer [21,52]. In the same study 200 mg single dose produced a mean C_max _of 104 ng/ml and mean area under curve (AUC) of 553 h ng/ml. The half-life of this dose was 2.6 ± 0.7 h. Interestingly a single dose of DIM alone caused a significant clinical improvement in patients with stage II and stage III cervical intraepithelial neoplasia (CIN) [21]. Administration of 2 mg/kg/day DIM via an oral route showed improvement in pap smear results, human papillomavirus (HPV) status, and improved CIN by 1 to 2 grades [21]. Several pharmacokinetic studies on DIM have stated that up to 300 mg in a single dose of DIM can be tolerated by humans [52], indicating that our dose of DIM falls within the accepted and tolerated dose. Cisplatin in our studies was given intraperitoneally at a dose of 5 mg/kg twice a week, which is 14 mg/m^2 ^when converted to the equivalent human dose [53]. The weekly dose of cisplatin ranges from 40 to 140 mg/m^2 ^in humans. We administered cisplatin twice a week, with an additive effect of 28 mg/m^2^, which is much lower than the regular dosage typically given to patients with cancer. If a low dose of cisplatin can be given to patients without the loss of any therapeutic effect but with reduced side effects, it would represent a significant breakthrough in clinical practice. Nevertheless, further clinical studies are needed to show that DIM can reduce the side effects of cisplatin.

In conclusion, our results firmly establish that DIM induces apoptosis in ovarian cancer cells by inhibiting STAT3 signaling. Our results also provide evidence that DIM inhibits the invasion of ovarian cancer cells and angiogenesis by inhibiting HIF-1α and VEGF, which are regulated by STAT3. Importantly, DIM potentiated the effect of cisplatin both in culture and *in vivo *by inhibiting STAT3. Taken together, the findings from our study provide support for the use of DIM alone or in combination with cisplatin in preclinical and clinical settings in the management of ovarian cancer patients.

## Conclusions

For the first time, our study demonstrates that DIM targets STAT3 to suppress the growth of ovarian tumor cells *in vitro *and *in vivo*. Importantly, DIM inhibits angiogenesis and invasion of ovarian cancer cells. Our results also show that targeting STAT3 potentiates the effect of cisplatin both in culture as well as in a tumor xenograft model.

## Competing interests

The authors declare that they have no competing interests.

## Authors' contributions

Both PKK and SKS were responsible for designing the study, analyzing the data and writing the manuscript. Experiments were performed by PKK. Both authors read and approved the final manuscript.

## Pre-publication history

The pre-publication history for this paper can be accessed here:

http://www.biomedcentral.com/1741-7015/10/9/prepub

## References

[B1] Jemal A Siegel R Xu J Ward E Cancer statistics, 2010 CA Cancer J Clin 2010 60 277 300 10.3322/caac.20073 20610543

[B2] Ozols RF Bookman MA du Bois A Pfisterer J Reuss A Young RC Intraperitoneal cisplatin therapy in ovarian cancer: comparison with standard intravenous carboplatin and paclitaxel Gynecol Oncol 2006 103 1 6 16904166 10.1016/j.ygyno.2006.06.026

[B3] Sledge GW Jr Loehrer PJ Sr Roth BJ Einhorn LH Cisplatin as first-line therapy for metastatic breast cancer J Clin Oncol 1988 6 1811 1814 3199166 10.1200/JCO.1988.6.12.1811

[B4] Sheikh-Hamad D Timmins K Jalali Z Cisplatin-induced renal toxicity: possible reversal by N-acetylcysteine treatment J Am Soc Nephrol 1997 8 1640 1644 9335396 10.1681/ASN.V8101640

[B5] Duan Z Foster R Bell DA Mahoney J Wolak K Vaidya A Hampel C Lee H Seiden MV Signal transducers and activators of transcription 3 pathway activation in drug-resistant ovarian cancer Clin Cancer Res 2006 12 5055 5063 10.1158/1078-0432.CCR-06-0861 16951221

[B6] Horvath CM STAT proteins and transcriptional responses to extracellular signals Trends Biochem Sci 2000 25 496 502 10.1016/S0968-0004(00)01624-8 11050435

[B7] Horvath CM The Jak-STAT pathway stimulated by interleukin 6 Sci STKE 2004 2004 tr9 10.1126/stke.2602004tr9 15561981

[B8] Epling-Burnette PK Liu JH Catlett-Falcone R Turkson J Oshiro M Kothapalli R Li Y Wang JM Yang-Yen HF Karras J Jove R Loughran TP Jr Inhibition of STAT3 signaling leads to apoptosis of leukemic large granular lymphocytes and decreased Mcl-1 expression J Clin Invest 2001 107 351 362 10.1172/JCI9940 11160159 PMC199188

[B9] Gritsko T Williams A Turkson J Kaneko S Bowman T Huang M Nam S Eweis I Diaz N Sullivan D Yoder S Enkemann S Eschrich S Lee JH Beam CA Cheng J Minton S Muro-Cacho CA Jove R Persistent activation of stat3 signaling induces survivin gene expression and confers resistance to apoptosis in human breast cancer cells Clin Cancer Res 2006 12 11 19 10.1158/1078-0432.CCR-04-1752 16397018

[B10] Huang M Page C Reynolds RK Lin J Constitutive activation of stat 3 oncogene product in human ovarian carcinoma cells Gynecol Oncol 2000 79 67 73 10.1006/gyno.2000.5931 11006034

[B11] Niu G Wright KL Huang M Song L Haura E Turkson J Zhang S Wang T Sinibaldi D Coppola D Heller R Ellis LM Karras J Bromberg J Pardoll D Jove R Yu H Constitutive Stat3 activity up-regulates VEGF expression and tumor angiogenesis Oncogene 2002 21 2000 2008 10.1038/sj.onc.1205260 11960372

[B12] Wincewicz A Koda M Sulkowska M Kanczuga-Koda L Wincewicz D Sulkowski S STAT3 and hypoxia induced proteins--HIF-1alpha, EPO and EPOR in relation with Bax and Bcl-xL in nodal metastases of ductal breast cancers Folia Histochem Cytobiol 2009 47 425 430 10.2478/v10042-009-0099-7 20164027

[B13] Jung JE Lee HG Cho IH Chung DH Yoon SH Yang YM Lee JW Choi S Park JW Ye SK Chung MH STAT3 is a potential modulator of HIF-1-mediated VEGF expression in human renal carcinoma cells FASEB J 2005 19 1296 1298 15919761 10.1096/fj.04-3099fje

[B14] Rosen DG Mercado-Uribe I Yang G Bast RC Jr Amin HM Lai R Liu J The role of constitutively active signal transducer and activator of transcription 3 in ovarian tumorigenesis and prognosis Cancer 2006 107 2730 2740 10.1002/cncr.22293 17063503

[B15] Ciska E Verkerk R Honke J Effect of boiling on the content of ascorbigen, indole-3-carbinol, indole-3-acetonitrile, and 3,3'-diindolylmethane in fermented cabbage J Agric Food Chem 2009 57 2334 2338 10.1021/jf803477w 19292468

[B16] Bosetti C Negri E Franceschi S Pelucchi C Talamini R Montella M Conti E La Vecchia C Diet and ovarian cancer risk: a case-control study in Italy Int J Cancer 2001 93 911 915 10.1002/ijc.1422 11519057

[B17] Kandala PK Srivastava SK Activation of checkpoint kinase 2 by 3,3'-diindolylmethane is required for causing G2/M cell cycle arrest in human ovarian cancer cells Mol Pharmacol 2010 78 297 309 10.1124/mol.110.063750 20444961 PMC2917853

[B18] Chang X Tou JC Hong C Kim HA Riby JE Firestone GL Bjeldanes LF 3,3'-Diindolylmethane inhibits angiogenesis and the growth of transplantable human breast carcinoma in athymic mice Carcinogenesis 2005 26 771 778 10.1093/carcin/bgi018 15661811

[B19] Banerjee S Wang Z Kong D Sarkar FH 3,3'-Diindolylmethane enhances chemosensitivity of multiple chemotherapeutic agents in pancreatic cancer Cancer Res 2009 69 5592 5600 10.1158/0008-5472.CAN-09-0838 19531648 PMC2743468

[B20] Rahman KW Sarkar FH Inhibition of nuclear translocation of nuclear factor-κB contributes to 3,3'-diindolylmethane-induced apoptosis in breast cancer cells Cancer Res 2005 65 364 371 15665315

[B21] Del Priore G Gudipudi DK Montemarano N Restivo AM Malanowska-Stega J Arslan AA Oral diindolylmethane (DIM): pilot evaluation of a nonsurgical treatment for cervical dysplasia Gynecol Oncol 2010 116 464 467 10.1016/j.ygyno.2009.10.060 19939441

[B22] Heath EI Heilbrun LK Li J Vaishampayan U Harper F Pemberton P Sarkar FH A phase I dose-escalation study of oral BR-DIM (BioResponse 3,3'- Diindolylmethane) in castrate-resistant, non-metastatic prostate cancer Am J Transl Res 2010 2 402 411 20733950 PMC2923864

[B23] Banerjee S Kong D Wang Z Bao B Hillman GG Sarkar FH Attenuation of multi-targeted proliferation-linked signaling by 3,3'-diindolylmethane (DIM): from bench to clinic Mutat Res 2011 728 47 66 10.1016/j.mrrev.2011.06.001 21703360 PMC4120774

[B24] Auersperg N Siemens CH Myrdal SE Human ovarian surface epithelium in primary culture In Vitro 1984 20 743 755 10.1007/BF02618290 6083974

[B25] Boreddy SR Pramanik KC Srivastava SK Pancreatic tumor suppression by benzyl isothiocyanate is associated with inhibition of PI3K/AKT/FOXO pathway Clin Cancer Res 2011 17 1784 1795 10.1158/1078-0432.CCR-10-1891 21350002 PMC3076680

[B26] Batra S Sahu RP Kandala PK Srivastava SK Benzyl isothiocyanate-mediated inhibition of histone deacetylase leads to NF-kappaB turnoff in human pancreatic carcinoma cells Mol Cancer Ther 2010 9 1596 1608 10.1158/1535-7163.MCT-09-1146 20484017 PMC2946330

[B27] Sahu RP Srivastava SK The role of STAT-3 in the induction of apoptosis in pancreatic cancer cells by benzyl isothiocyanate J Natl Cancer Inst 2009 101 176 193 10.1093/jnci/djn470 19176463 PMC2724856

[B28] Pyun BJ Choi S Lee Y Kim TW Min JK Kim Y Kim BD Kim JH Kim TY Kim YM Kwon YG Capsiate, a nonpungent capsaicin-like compound, inhibits angiogenesis and vascular permeability via a direct inhibition of Src kinase activity Cancer Res 2008 68 227 235 10.1158/0008-5472.CAN-07-2799 18172315

[B29] Cho SG Yi Z Pang X Yi T Wang Y Luo J Wu Z Li D Liu M Kisspeptin-10, a KISS1-derived decapeptide, inhibits tumor angiogenesis by suppressing Sp1-mediated VEGF expression and FAK/Rho GTPase activation Cancer Res 2009 69 7062 7070 10.1158/0008-5472.CAN-09-0476 19671799 PMC3242001

[B30] Burke WM Jin X Lin HJ Huang M Liu R Reynolds RK Lin J Inhibition of constitutively active Stat3 suppresses growth of human ovarian and breast cancer cells Oncogene 2001 20 7925 7934 10.1038/sj.onc.1204990 11753675

[B31] Anglesio MS George J Kulbe H Friedlander M Rischin D Lemech C Power J Coward J Cowin PA House CM Chakravarty P Gorringe KL Campbell IG Australian Ovarian Cancer Study Group Okamoto A Birrer MJ Huntsman DG de Fazio A Kalloger SE Balkwill F Gilks CB Bowtell DD IL6-STAT3-HIF signaling and therapeutic response to the angiogenesis inhibitor sunitinib in ovarian clear cell cancer Clin Cancer Res 2011 17 2538 2548 10.1158/1078-0432.CCR-10-3314 21343371

[B32] Syed V Ulinski G Mok SC Ho SM Reproductive hormone-induced, STAT3-mediated interleukin 6 action in normal and malignant human ovarian surface epithelial cells J Natl Cancer Inst 2002 94 617 629 10.1093/jnci/94.8.617 11959895

[B33] Wei D Le X Zheng L Wang L Frey JA Gao AC Peng Z Huang S Xiong HQ Abbruzzese JL Xie K Stat3 activation regulates the expression of vascular endothelial growth factor and human pancreatic cancer angiogenesis and metastasis Oncogene 2003 22 319 329 12545153 10.1038/sj.onc.1206122

[B34] Colomiere M Ward AC Riley C Trenerry MK Cameron-Smith D Findlay J Ackland L Ahmed N Cross talk of signals between EGFR and IL-6R through JAK2/STAT3 mediate epithelial-mesenchymal transition in ovarian carcinomas Br J Cancer 2009 100 134 144 10.1038/sj.bjc.6604794 19088723 PMC2634691

[B35] Bromberg JF Wrzeszczynska MH Devgan G Zhao Y Pestell RG Albanese C Darnell JE Jr Stat3 as an oncogene Cell 1999 98 295 303 10.1016/S0092-8674(00)81959-5 10458605

[B36] Buettner R Mora LB Jove R Activated STAT signaling in human tumors provides novel molecular targets for therapeutic intervention Clin Cancer Res 2002 8 945 954 11948098

[B37] Bromberg J Darnell JE Jr The role of STATs in transcriptional control and their impact on cellular function Oncogene 2000 19 2468 2473 10.1038/sj.onc.1203476 10851045

[B38] Bowman T Garcia R Turkson J Jove R STATs in oncogenesis Oncogene 2000 19 2474 2488 10.1038/sj.onc.1203527 10851046

[B39] Amin HM McDonnell TJ Ma Y Lin Q Fujio Y Kunisada K Leventaki V Das P Rassidakis GZ Cutler C Medeiros LJ Lai R Selective inhibition of STAT3 induces apoptosis and G(1) cell cycle arrest in ALK-positive anaplastic large cell lymphoma Oncogene 2004 23 5426 5434 10.1038/sj.onc.1207703 15184887

[B40] Huang S Sinicrope FA Sorafenib inhibits STAT3 activation to enhance TRAIL-mediated apoptosis in human pancreatic cancer cells Mol Cancer Ther 2010 9 742 750 10.1158/1535-7163.MCT-09-1004 20197401 PMC3281304

[B41] Rahman KW Li Y Wang Z Sarkar SH Sarkar FH Gene expression profiling revealed survivin as a target of 3,3'-diindolylmethane-induced cell growth inhibition and apoptosis in breast cancer cells Cancer Res 2006 66 4952 4960 10.1158/0008-5472.CAN-05-3918 16651453

[B42] Asechi H Hatano E Nitta T Tada M Iwaisako K Tamaki N Nagata H Narita M Yanagida A Ikai I Uemoto S Resistance to cisplatin-induced apoptosis via PI3K-dependent survivin expression in a rat hepatoma cell line Int J Oncol 2010 37 89 96 20514400

[B43] Chock KL Allison JM Shimizu Y El Shamy WM BRCA1-IRIS overexpression promotes cisplatin resistance in ovarian cancer cells Cancer Res 2010 70 8782 8791 10.1158/0008-5472.CAN-10-1352 20940403

[B44] Bryant CS Munkarah AR Kumar S Batchu RB Shah JP Berman J Morris RT Jiang ZL Saed GM Reduction of hypoxia-induced angiogenesis in ovarian cancer cells by inhibition of HIF-1 alpha gene expression Arch Gynecol Obstet 2010 282 677 683 10.1007/s00404-010-1381-9 20140681

[B45] Leong H Mathur PS Greene GL Green tea catechins inhibit angiogenesis through suppression of STAT3 activation Breast Cancer Res Treat 2009 117 505 515 10.1007/s10549-008-0196-x 18821062 PMC3664280

[B46] Hess LM Benham-Hutchins M Herzog TJ Hsu CH Malone DC Skrepnek GH Slack MK Alberts DS A meta-analysis of the efficacy of intraperitoneal cisplatin for the front-line treatment of ovarian cancer Int J Gynecol Cancer 2007 17 561 570 10.1111/j.1525-1438.2006.00846.x 17504373

[B47] Hoskins WJ Prospective on ovarian cancer: why prevent? J Cell Biochem Suppl 1995 23 189 199 8747396 10.1002/jcb.240590926

[B48] Parker RJ Eastman A Bostick-Bruton F Reed E Acquired cisplatin resistance in human ovarian cancer cells is associated with enhanced repair of cisplatin-DNA lesions and reduced drug accumulation J Clin Invest 1991 87 772 777 10.1172/JCI115080 1999494 PMC329864

[B49] Whiteside MA Piyathilake CJ Bushell TM Johanning GL Intrinsic cisplatin resistance in lung and ovarian cancer cells propagating in medium acutely depleted of folate Nutr Cancer 2006 54 274 284 10.1207/s15327914nc5402_14 16898872

[B50] Selvendiran K Bratasz A Tong L Ignarro LJ Kuppusamy P NCX-4016, a nitro-derivative of aspirin, inhibits EGFR and STAT3 signaling and modulates Bcl-2 proteins in cisplatin-resistant human ovarian cancer cells and xenografts Cell Cycle 2008 7 81 88 10.4161/cc.7.1.5103 18196976 PMC2890223

[B51] Grose KR Bjeldanes LF Oligomerization of indole-3-carbinol in aqueous acid Chem Res Toxicol 1992 5 188 193 10.1021/tx00026a007 1643248

[B52] Reed GA Sunega JM Sullivan DK Gray JC Mayo MS Crowell JA Hurwitz A Single-dose pharmacokinetics and tolerability of absorption-enhanced 3,3'-diindolylmethane in healthy subjects Cancer Epidemiol Biomarkers Prev 2008 17 2619 2624 10.1158/1055-9965.EPI-08-0520 18843002 PMC2602858

[B53] Reagan-Shaw S Nihal M Ahmad N Dose translation from animal to human studies revisited FASEB J 2008 22 659 661 17942826 10.1096/fj.07-9574LSF

